# Spatial Segregation within the Spawning Migration of North Eastern Atlantic Mackerel (*Scomber scombrus*) as Indicated by Juvenile Growth Patterns

**DOI:** 10.1371/journal.pone.0058114

**Published:** 2013-02-28

**Authors:** Teunis Jansen, Andrew Campbell, Thomas Brunel, Lotte Worsøe Clausen

**Affiliations:** 1 DTU AQUA – National Institute of Aquatic Resources, Technical University of Denmark, Charlottenlund, Denmark; 2 Fisheries Ecosystems Advisory Services, Marine Institute, Galway, Ireland; 3 Fisheries Department, IMARES, Ijmuiden, Nederland; Swansea University, United Kingdom

## Abstract

A comparison of growth data (fish length) with latitude shows that southern juvenile mackerel attain a greater length than those originating from further north before growth ceases during their first winter. A similar significant relationship was found between the growth in the first year (derived from the otolith inner winter ring) and latitude for adult mackerel spawning between 44°N (Bay of Biscay) and 54°N (west of Ireland). These observations are consistent with spatial segregation of the spawning migration; the further north that the fish were hatched, the further north they will tend to spawn. No such relationship was found in mackerel spawning at more northerly latitudes, possibly as a consequence of increased spatial mixing in a more energetic regime with stronger currents. This study provides previously lacking support for spawning segregation behaviour among North East Atlantic mackerel – an important step towards understanding the migratory behaviour of mackerel and hence the spatiotemporal distribution dynamics around spawning time.

## Introduction

Mackerel (*Scomber scombrus*) is one of the most abundant and widely distributed migratory fish species in the North East Atlantic [1]. Knowledge of the population structure of an exploited fish species is key to understanding its basic population biology and a necessary prerequisite for providing effective advice to fisheries managers. The population structure of mackerel has consequently been the subject of repeated studies over the last 100 years of mackerel science [2], [3].

The North Eastern Atlantic mackerel (NEAM) stock has traditionally been divided into 3 separate spawning components; a southern, western and North Sea component [4]. NEAM mainly spawn on the continental shelf from Biscay in the south to the west of Scotland and in the North Sea. While the southern and western spawning areas are connected, the North Sea area is spatially separated by reduced spawning in the English and Fair Isle channels [5]. Most studies on natal homing have focused on identifying differences between mackerel in the North Sea and in the west. The results of such studies have, to date, failed to conclusively demonstrate natal homing in these stock components. While the initial analyses were based on landing statistics [3], more recent approaches have attempted to distinguish between individuals based on geno/phenotypic classification. Some studies on characteristics such as juvenile growth patterns in otoliths [6], [7], protein polymorphism [8], [9] and tapeworm (*Grillotia smarisgora*) infection rates [10] were based on individuals from the respective spawning areas that were not all in the process of spawning (i.e. ripe/running). These studies may have included mackerel from several discrete components, due to the swimming capabilities of mackerel. After spawning, some mackerel from the south-western areas of the NEA, migrate into the North Sea before spawning in the North Sea has ceased [11]. Consequently, conclusions on natal homing and the existence of multiple components cannot be drawn from these studies. Other studies were correctly based on spawning individuals, but found no difference in ectoparasite infections [12], blood phenotypes [13], allozymes [8] and (unlike in the west Atlantic [14]), otolith shapes (Jansen unpubl. found no significant differences between spawning components in an analysis of 652 spawning mackerel). In recent years, modern genetic approaches have been applied, but with inconclusive results. While mitochondrial DNA from relatively few spawning mackerel did not group into the expected clades, statistical analysis of the same allele frequencies separated the 3 western samples from the rest providing some, albeit weak, support for genetic differentiation on an ecological time scale [15]. A more recent work on mackerel genetics does not support a separation. In conclusion, there is presently no support for the hypothesis of multiple separate natal homing components /stocks/contingents (referred to as ‘contingents’ herein) within the wider NEAM population.

In this study, we examine the spawning migration by following spatially related growth patterns from early life to spawning adults in the North East Atlantic. Key factors affecting somatic growth of mackerel larvae and juveniles may vary with latitude throughout the wide spawning area. The length increment during the first year of growth can be postulated to be dependent on the date of birth, and on the growth rate, which is influenced by ambient temperature and food availability. Since mackerel spawn earlier at southern latitudes, the first growth season is longer for southern mackerel. Also, ambient temperatures are generally higher at southern latitudes, allowing for faster growth rate, but also higher energy requirements. Given that NEA mackerel does not initiate size dependent migratory behaviour prior to maturation [11], we can expect the body-size of juvenile mackerel to be negatively correlated with latitude at the end of the first growth season, a relationship that has not been previously demonstrated for mackerel. We further examine whether this spatial pattern of first year growth are preserved through all ages. This is achieved through measurements of otolith growth from hatch to first winter. Otolith growth during the first growth season is used as a proxy for somatic growth based on the strong correlation between mackerel body length and otolith length at the end of the first growth season [7]. The structural properties of otolith growth and formation allows for measurement of the first growth zone in mackerel of any age.

## Materials and Methods

### Mackerel length growth

Mean body length by quarter and ICES subarea were used as reported annually to the International Council for Exploration of the Sea (ICES). The data were obtained from tables in the annual mackerel assessment reports (*e.g.* [1]). Samples originate from both commercial fisheries and scientific surveys. Only data from the first quarter (January-March) were used in the final analysis because initial data exploration indicated that growth had not ceased in the fourth quarter (October-December). The available length observations were averaged by stratum, i.e. by year, quarter, ICES subarea and country by the individual national sampling programs before reporting to ICES. While length observations are usually expected to be log-gaussian distributed, we could assume that mean length (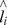) in stratum 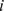 has a gaussian distribution with a mean vector
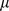
and standard deviation
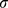
due to the relatively high number of observations (mean  = 2455):
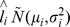



We examined the growth-latitude relation by modelling length with the following predictor variables:

- *Latitude* (considered to be the center of the appropriate ICES subarea).
- *Spawning component* ( =  North Sea or South-west). Mackerel in the North Sea might be a separate spawning component [1], [16].
- *Year*. Including a year effect permits interannual environmental variation (food, temperature, currents etc.) to be considered.

Since mean length can only be positive, we expressed the systematic effect as:
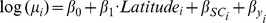
where
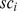
is the spawning component and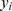 is the year of the 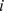
'th observation.

The lack of individual length observations complicated the error term in the model. We addressed this by modeling the variance as the sum of the individual errors due to sampling (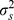) and model implementation (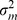). Since the variance of the mean is equivalent to the variance of the individual observations divided by the number of averaged observations, the variance of a mean length in stratum 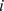 can be written:
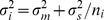



Which leads to the following model formulation:
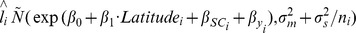



The *corvif* function of the AED R-package was used to calculate Variance Inflation Factors (VIF). VIFs are indicators of collinearity. The predictor variables were sufficiently independent to be used in the same model fit, if the VIFs were >3 [17]. Model parameters 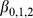, 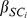, 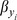, 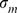
and 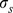 were estimated using the Nelder-Mead method for identifying the maximum likelihood model.

In order to ensure that a latitude-length relation at the end of the first growth season is not a consequence of size-dependent migration, we also explored the variability of this relationship through the first two years of growth.

### Mackerel otolith growth

Sagittal otoliths extracted from mackerel caught by commercial vessels and scientific surveys in 2002–2003 and 2005–2006 were examined. The archived otoliths were embedded in resin (histokitt). Otoliths were viewed in reflected light under a stereo microscope (Leica MZ6) and images digitised (Leica DFC320 camera and Leica IM 50 frame grabber) using a standard setup of exposure (107.9 ms, 8 bits/channel with a frame of 1300×1030 pixels), light intensity, angle and direction of illumination. The length of the first winter ring (L1) was measured as the distance between the anterior and posterior centres of the first broad opaque band (Table S1. Start model and parameter estimate for final models based on the entire area. Figure S1) from otoliths taken from spawning mackerel (ICES maturity stage 6 *i.e.* “ripe or running”) from 2–11 years of age.

We subsequently examined the growth-latitude relation by modelling L1 with the following predictor variables:

- *Latitude* (numeric).
- *Spawning component* (Factor: North Sea/South-west). Mackerel in the North Sea might be a separate spawning component [1], [16].
- *Year class* (Factor: Year – age). This year specific effect represents the interannual variation of environmental parameters that can affect growth rates (food, temperature, currents etc.).
- *Day of year* (Numeric). To account for seasonal effects.
- *Total body length* (numeric). To account for size based effects.
- 2^nd^ order interactions.

Variance Inflation Factors (VIF) were calculated for the predictor variables to ensure that the model output was not affected by collinearity (VIF >3) [17]. Multivariate linear regression modeling was done “backwards” by sequentially removing insignificant (p>0.05) terms starting with 2^nd^ order interactions.

The modelling was performed using R statistical software (version 2.12.1) incorporating the “stats”, “bbmle”, “nlme”, “nortest” and “AED” packages [18].

## Results

The body length dataset consisted of 132 records of mean length by ICES subarea derived from 366,570 individual length observations. The available samples cover all ICES sub areas throughout the spawning and nursery areas from the Bay of Biscay to the North Sea region, over the period 1997 to 2010. The only significant term in the model of mackerel body length at the end of the first growth season was *latitude*.

**Figure 1:**
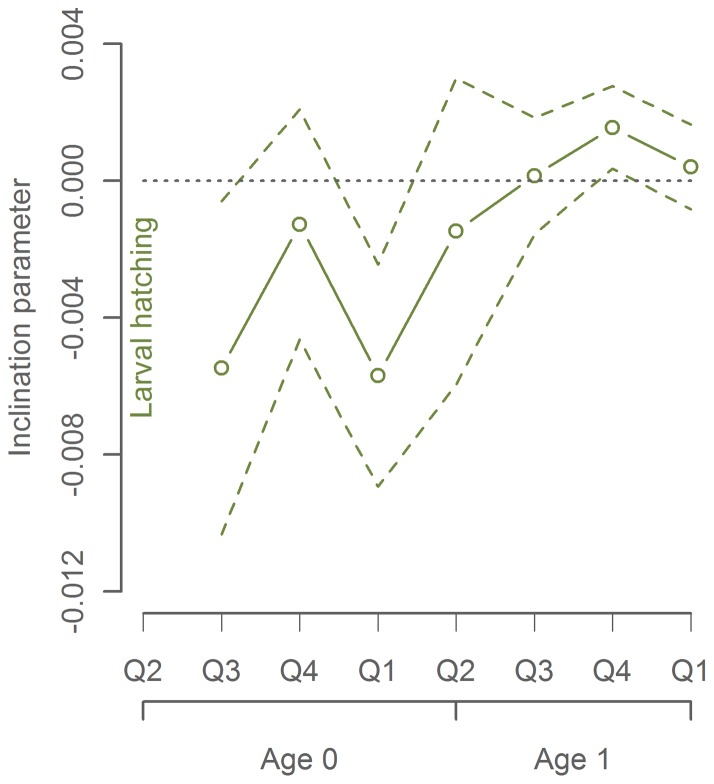
Latitude-length relation by mackerel age. Early life observations during the first year show negative latitude-length relations (p = 0.0011), while later observations show no significant correlation. The inclination (slope) parameter (*β_1_*) on the Y-axis are the effect of latitude in the model log(*mean body length*)  =  *β_0_* + *β_1_* * *Latitude* + *β_y_* + *ε*, where *β_y_* is the year-specific effect and *ε* is the random error term.

In order to ensure that the observed relationship between latitude and body length after the first year of growth is not a consequence of size- dependent migration, we investigated the variability of this relationship through the first two years of growth. While the earliest observations (July-September) confirmed the negative latitude-length relation, later observations (during the mackerel's second year) showed no significant correlation, consistent with spatial mixing (Figure 1).

**Figure 2:**
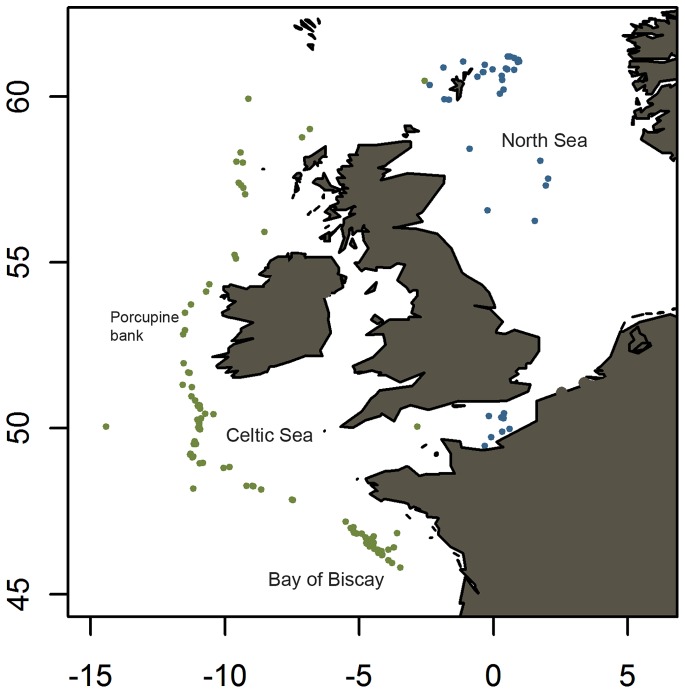
Otolith sample locations and geographical names referred to in the text. Blue circles mark sample locations of North Sea mackerel, green circles mark sample locations of western mackerel.

The L1 dataset (first winter ring) comprised 1,265 individual measurements with samples broadly distributed throughout the spawning area from the northern Bay of Biscay to the North Sea region (Figure 2). As before, the only significant term in the model of L1 was *latitude* (p<0.001, Table S1).

**Figure 3:**
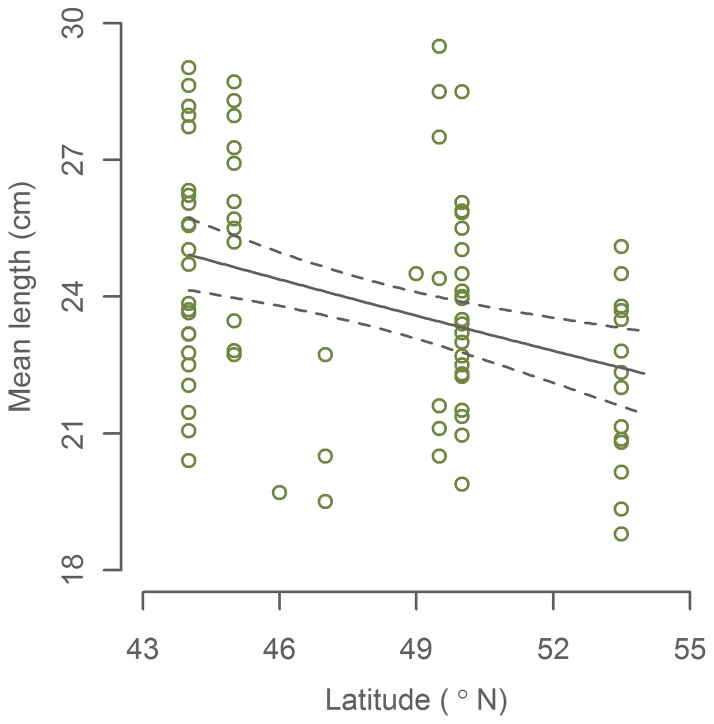
Mean body length of mackerel at the end of the first growth season (January-March) by latitude. 95% confidence interval of the fitted model is indicated by dashed lines. Data from 44–54°N.

**Figure 4:**
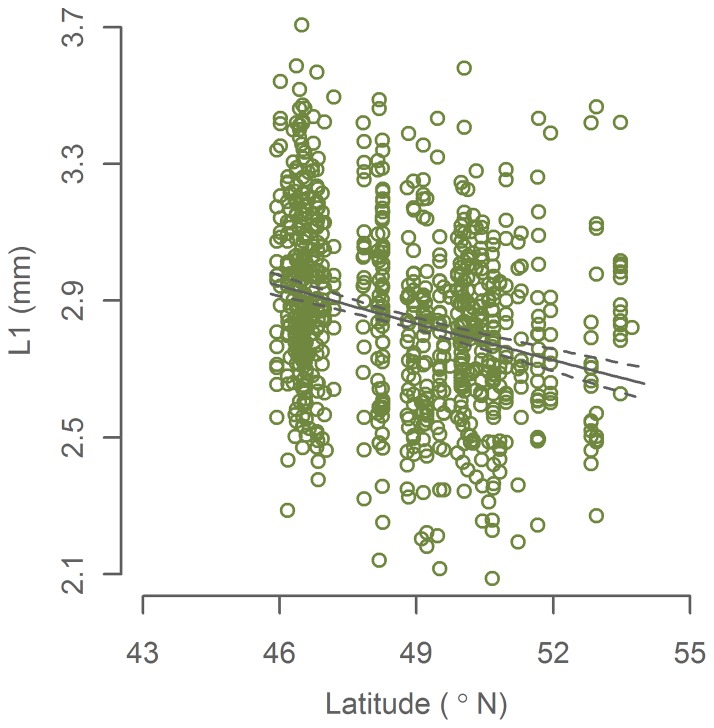
L1 (otolith growth from hatch to first winter) from spawning mackerel by latitude. 95% confidence interval of the fitted model is indicated by dashed lines. Data from 44–54°N.

Inspection of the model residuals in both growth-latitude models revealed that the negative correlations were mainly evident from 44°N to around 54°N (Figure S2, S3). In order to explore this spatial pattern, we repeated the modelling steps separately for two geographical regions: 44°N –54°N and 54°N –61.2°N. While no significant terms were found in the northern models (p>0.05), we again found *latitude* to be the only significant term in the southern models (Tables 1, 2, Figure 3, 4). Latitude was negatively correlated with body size and L1 with an estimated decrease of 9±5% and 10±3% over the 8.5° from the Bay of Biscay to west of Ireland. The residuals of both models were normal distributed (Anderson-Darling normality test: p>0.05) and do not display any distinct patterns.

**Table 1:** Parameter estimates for final model based on the area 44–54°N.

Significant terms	Est.	S.E.	P
Intercept	3.73	0.14	<0.001
Latitude	−0.012	0.003	<0.001

Start model: Log (Body length) ∼ factor(Year) +Latitude + E/W.

**Table 2:** Parameter estimates for final models based on the area 44–54°N.

Significant terms	Est.	S.E.	P
Intercept	1.67	0.08	<0.001
Latitude	−0.013	0.002	<0.001

Start model: Log (L1) ∼ factor(Year class) + DayOfYear + Latitude + E/W + Day: Lat interaction + E/W: YC interaction.

## Discussion

Our analyses demonstrate that, compared across latitudes, southern mackerel reach a larger size before growth ceases during their first winter. We found this relationship between latitude and juvenile growth in the first year to be significant not only for juveniles of age 1, but also preserved in adults that return to spawn between the Bay of Biscay in the south and west of Ireland in the north (44°N –54°N). These observations are consistent with a spatial segregation of the spawning migration, meaning that mackerel tend to return to spawn at higher latitudes compared with other individuals that were hatched more southerly.

The latitude-growth relationships from our study were statistically significant only in the area from the Biscay to west of Ireland. This area is partly a retention area, with currents that do not transport larvae and juveniles away, and partly an area with weak northwards flow along the shelf edge, which reduces spatial mixing and thus preserves the growth rate patterns in the population. This is in contrast to the areas further north where the strong north Atlantic current hits the European shelf edge around Porcupine bank and turns northwards on and along the shelf edge [19]. Mixing of larvae and juveniles from different spawning locations is therefore a likely explanation for the lack of spatial growth patterns in the area north of Porcupine bank. However, it is also possible that there is no latitude gradient in growth rates in this area.

The observed correlation between latitude and juvenile growth may be a result of several processes [7], . Lower temperatures and shorter growth seasons at higher latitudes [21] can be expected to result in lower growth rates [22], [23]. Size-seasonal-specific mortality may also have an effect. Size-seasonal-specific mortality patterns can emerge because the higher metabolic and growth rate as experienced in the warmer southern waters results in a need to feed at a higher rate in order to keep up with the elevated energy losses. Larvae that are unable to find sufficient nutrients subsequently starve and die. This is more pronounced in warmer than in cooler waters and could theoretically lead to the patterns observed in the present study. The principal conclusion of the study relies only on the observed correlation between growth and latitude – it is independent of the actual causal effects.

In the case that the tendency for spatially segregated spawning of North East Atlantic mackerel had been sufficiently strong over an evolutionary time scale, then it should have led to genetic differentiation. However, previous studies have indicated a weak or complete lack of genetic differentiation. This suggests that, on an evolutionary time scale, the rate of mixing has been too high for genetic differences to become apparent. Further work on the balance between isolation and mixing and the effects on genetic differentiation on long term evolutionary vs. short term ecological time scales is needed.

The North Sea mackerel have traditionally been considered as a separate stock because spawning in the North Sea is spatially separated from that of the western and southern components and because of a significant local depletion that has been interpreted as a stock collapse [16], [24]. The lack of subsequent rebuilding in the North Sea during decades of high abundance in the southern/western areas could indicate isolation. The growth patterns we observed were all within the western spawning component. If the spawning migration of mackerel originating from different areas within one spawning component is spatially segregated, then it seems reasonable to expect an even more pronounced segregation between spawning components. However, the present analysis did not provide the opportunity to show separate growth patterns in the North Sea and in the areas west of Scotland.

This study provides the first support for spawning segregation behaviour among North East Atlantic mackerel – an important step towards understanding the migratory behaviour of mackerel and hence the spatiotemporal distribution dynamics around spawning time.

## Supporting Information

Figure S1 **Sagittal otolith showing L1 (otolith growth from hatch to first winter).**
(TIFF)

Figure S2 **Mean body length of mackerel at the end of the first growth season (January-March) by latitude (including 54–61°N).**
(TIF)

Figure S3 **L1 (otolith growth from hatch to first winter) from spawning mackerel by latitude (including 54–61°N).**
(TIF)

Table S1 **Start model and parameter estimate for final models based on the entire area.**
(DOCX)
