# Supplementary figures and images for: Spatial Segregation within the Spawning Migration of North Eastern Atlantic Mackerel (Scomber scombrus) as Indicated by Juvenile Growth Patterns
Source: PLoS One. 2013 Feb 28;8(2):e58114. doi: 10.1371/journal.pone.0058114 (PMC3585244; doi:10.1371/journal.pone.0058114)

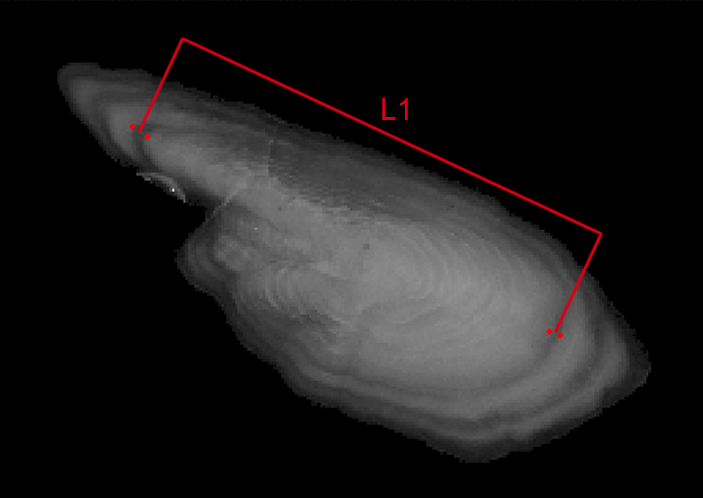

Supplement: Figure S1 — Sagittal otolith showing L1 (otolith growth from hatch to first winter). (TIFF) [file pone.0058114.s001.tiff]

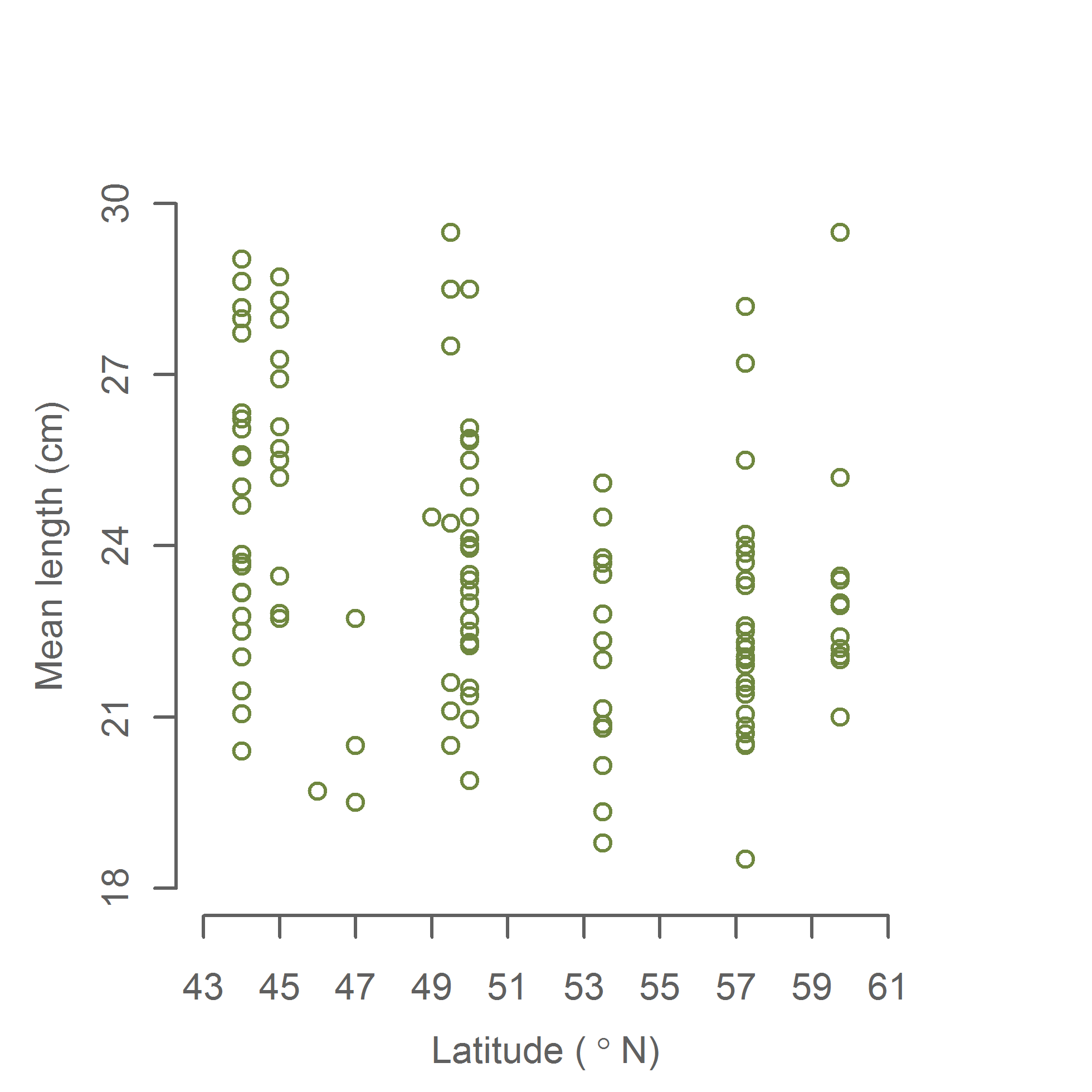

Supplement: Figure S2 — Mean body length of mackerel at the end of the first growth season (January-March) by latitude (including 54–61°N). (TIF) [file pone.0058114.s002.tif]

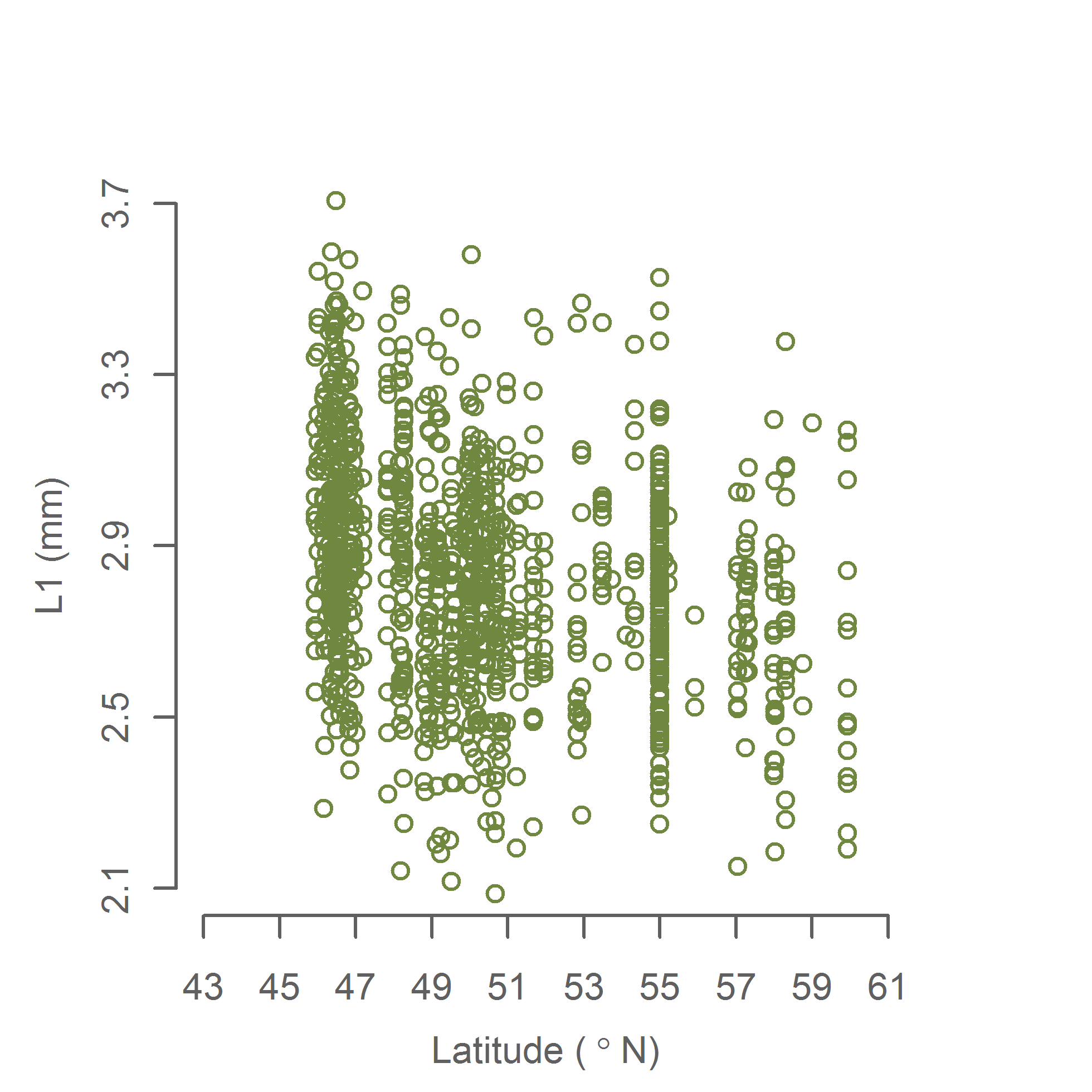

Supplement: Figure S3 — L1 (otolith growth from hatch to first winter) from spawning mackerel by latitude (including 54–61°N). (TIF) [file pone.0058114.s003.tif]
