# Supplementary material for: Spatial Segregation within the Spawning Migration of North Eastern Atlantic Mackerel (Scomber scombrus) as Indicated by Juvenile Growth Patterns
Source: PLoS One. 2013 Feb 28;8(2):e58114. doi: 10.1371/journal.pone.0058114 (PMC3585244; doi:10.1371/journal.pone.0058114)
Supplement: Table S1 — Start model and parameter estimate for final models based on the entire area. (DOCX) [file pone.0058114.s004.docx]

| Log (L1) ~  factor(Year class) +  DayOfYear +  Latitude +  E/W +  Day : Lat interaction + E/W : YC interaction | Intercept | 3.89 | 0.11 | <0.001 |
| --- | --- | --- | --- | --- |
|  | Latitude | -0.02 | 0.002 | <0.001 |
